# Notes on a complicated relationship: scientific pluralism, epistemic relativism, and stances

**DOI:** 10.1007/s11229-020-02943-2

**Published:** 2020-11-09

**Authors:** Sophie Juliane Veigl

**Affiliations:** Cohn Institute for the History and Philosophy of Science and Ideas, Humanities Faculty, Tel Aviv University, Ramat Aviv, Tel Aviv, 6997801 Israel

**Keywords:** Epistemic relativism, Scientific pluralism, Renunciation of judgment, Stances

## Abstract

While scientific pluralism enjoys widespread popularity within the philosophy of science, a related position, epistemic relativism, does not have much traction. Defenders of scientific pluralism, however, dread the question of whether scientific pluralism entails epistemic relativism. It is often argued that if a scientific pluralist accepts epistemic relativism, she will be unable to pass judgment because she believes that “anything goes”. In this article, I will show this concern to be unnecessary. I will also argue that common strategies to differentiate relativism and pluralism fail. Building upon this analysis, I will propose a new way of looking at both positions’ relations. This article aims to understand what explains the friction between scientific pluralism and epistemic relativism. I will demonstrate that conceptualizing both epistemic relativism and scientific pluralism as “stances” sheds better light on their relation and demonstrates that it is, in principle, possible to support both positions at the same time. Preferred policies and levels of analysis, however, cause friction in practice.

## Introduction

Historian of science Lorraine Daston recently observed that relativism is one of the “ghouls and goblins allegedly let loose” by Thomas Kuhn, which today barely elicits a yawn (Daston 2016, p. 119). This diagnosis is accurate for much of the contemporary philosophy of science. Whereas epistemic relativism (ER) does not enjoy great popularity amongst philosophers of science, another position, scientific pluralism (SP), certainly does.^1^ Defenders of SP are concerned about the question of whether SP entails ER. One intrinsic feature of almost every monograph defending a particular version of SP includes a section on why SP does not entail ER. Most of these arguments are quite similar. Card-carrying scientific pluralists argue that their position does not lead to ER because they do not renounce judgment (Kellert, Longino and Waters 2006; Mitchell 2003; Chang 2012). Their SP does not allow an “anything goes”-type of pluralism. They are not inclined to accept “any” theory (as equal). Their SP is, thus, immunized against the charge of ER. Card-carrying epistemic relativists, however, argue that they do not “renounce judgment” either but that judgment is preserved within the context of a particular epistemic system (ES) (Kusch 2020; Rovane 2011). What, thus, accounts for the differences between SP and ER^2^? Which aspects of ER and SP produce friction between both positions?

SP and ER are quite common notions within philosophy and the philosophy of science in particular. There are, however, many definitions of SP and ER, respectively. Let me thus immediately define what the terms ER and SP will denote in this paper. Philosopher and sociologist of science Martin Kusch provides a “standard model of relativism,” which surveys eight criteria collected from definitions given by friends and foes of relativism (Kusch 2016, p. 107f). I will adopt this model, with two minor changes (explicated in the footnotes).

1. Dependence: the epistemic status of a belief is relative to (a) an ES, or (b) a bundle of precedents, judged as virtuous;
2. Plurality^3^: there are, were, or might be several of such ES or bundles;
3. Non-Absolutism: none of these ES or bundles are absolutely warranted;
4. Exclusiveness: some of these ES or bundles can be exclusive (that is, incompatible), either because (a) they give different yes/no answers to the same questions (questions about the epistemic status of a belief) or because (b) they involve incommensurable forms of action;
5. Symmetry: ES or bundles are to be treated symmetrically because either (a) they are only based on local reasons for credibility, or (b) there is no neutral way of ranking them or (c) they are all equally good,^4^ or (d) the question of ranking does not arise for the proponents of each ES or bundle, or (e) to ensure successful sociological examination, different ES should be approached symmetrically, as a methodological choice;^5^
6. Conversion: switching from one ES to the other is not possible nor plausible by rational comparison;
7. Semantic Relativity: the statement “A subject S is epistemically justified believing p” expresses a proposition of either one of the two forms: (a) as per the ES or the bundle I (the speaker) am based on, is S epistemically justified to believe p (semantic contextualism) or (b) S is epistemically justified to believe p. This proposition is relatively false or true, depending on the contexts of assessment. (semantic relativism);
8. Metaphysical implications: (a) factual understanding: to be epistemically justified is a real relation between convictions and other factors. Epistemic relativism is the thesis that ES or bundles are a central component within this relation; (b) non-factual understanding: epistemic relativism is no metaphysical but a language-philosophical thesis.

For a definition of SP, I will rely on Steven Kellert’s, Helen Longino’s, and C. Kenneth Waters’ “pluralism-manifesto” *The Pluralist Stance* (2006). While not explicitly listing criteria for SP, the authors deem these three commitments essential:

- Non-monism: it is not known or denied that the nature of the world is such that it can, at least in principle, be completely described or explained by one comprehensive account.
- Plurality: there are, might be, should be (the verb be can have different modalities here) several theories, explanations, or methods to approach the same natural phenomena.
- Acceptance: plurality is favorable.

The question regarding the relation of SP and ER is asymmetrically discussed in the SP and ER literature, respectively. The issue of whether SP entails ER is prominently addressed by scientific pluralists. This is not the case for the ER literature. Several articles consider plurality or pluralism a component of ER (Boghossian 2007; Kusch 2016; Carter 2017). The idea, however, that SP does not entail ER because ER renounces judgment has not directly been addressed in the ER literature. What is clear, however, is that not all epistemic relativists subscribe to SP, or at least not to every version of it (see, e.g., Kusch 2015, Chang 2015). For example, Kusch (2015) rejects the normative consequences of Chang’s pluralism. The relation of both positions, therefore, needs to be reevaluated. It is essential to understand which aspects of both positions produce friction between ER and SP. Uncovering these spots will also bring us closer to understanding which versions of ER and SP could go together, which cannot, and why.

In this article, I aim to criticize the most common accounts regarding the differences between SP and ER and discuss a fresh perspective to uncover points of friction between ER and SP. As a result, this paper covers a wide range of topics. In his *Prolegomena*, Immanuel Kant argues that sometimes hammer and chisel, and at other times the etching needle is needed (Kant 1783/2004, p. 9). This article is of the former kind. While trying to provide an overview of the relation of SP and ER and drawing a “big picture,” I am well aware that more detailed work on the discussed topics, that is, work with the etching needle, will be needed in the future. With this paper, I aim to introduce some arguments that might start a refined discussion regarding the relation of SP and ER, a discussion that avoids the infamous “renunciation of judgment” argument and strives towards an elaborate and informative exchange. The core message is that considering the relation of SP and ER through the lens of “stances” helps to locate points of friction as disagreements regarding policy and levels of analysis.

I will proceed in five consecutive steps. The first two steps involve evaluating the scientific pluralist’s most common strategies to argue why SP and ER do not go together. On the one hand, scientific pluralists opine that ER “renounces judgment” and that SP does not. Some state, besides, that because the epistemic relativist believes all accounts are “equally valid,” it is impossible to accept ER and SP. On the other hand, the pluralist maintains that introducing a particular set of epistemic values preserves judgment and avoids ER. I will thus first introduce the problems attached to the “renunciation of judgment” (RoJ) and argue that the relativist does not renounce judgment. Understanding the differences between SP and ER by reference to RoJ, therefore, fails. Second, I will examine another strategy to differentiate SP and ER: the introduction of epistemic values. Several card-carrying scientific pluralists argue that their pluralism does not entail ER because they maintain the possibility to choose between theories. They preserve judgment through the introduction of epistemic values. I will argue that this strategy fails to clear SP of ER because epistemic values introduce relativism on the level of value choice.

Third, having shown that the two most common strategies to differentiate ER and SP fail, I will examine ER and SP in detail. I will argue that pluralists often do not support “symmetry” or “exclusiveness.” Relativists, on the other hand, might not support the pluralist’s “acceptance” or “non-monism.” Thus SP does not “entail” or “lead to” ER. There are, however, some forms of SP that are compatible with some forms of ER, but there simply is no one-dimensional relation between both positions.

In the fourth step, I will propose a different approach to the relation between ER and SP. Having established that there is no “one-fits-all” way of sketching the relation of both positions, I will move forward to understand the friction between SP and ER better. If

1. there is no reason why SP would entail ER, but there are versions of SP that are compatible with ER, and if
2. the most prominent “fears” of scientific pluralists regarding ER turn out to pose no threat ultimately,

then why are card-carrying scientific pluralists that concerned to maintain a boundary between SP and ER? I will put forward the idea that differences in commitments or preferred policies cause disagreement between pluralists and relativists. These differences also account for the intensity of the disagreements. If the main differences lie in commitments, values, emotions, and policies (VEPPs), looking at necessary and sufficient criteria might not be the best way to understand similarities and differences between SP and ER. I will argue, following Kusch (2020), that SP and ER should instead be viewed as “stances” in the van Fraassian meaning (2002), rather than as positions, comprised by necessary and sufficient criteria. This procedure connects this paper with a recent trend within the ER literature and the philosophy of science in general, which is to discuss the applicability of “stances” to several positions (Kusch 2020; Baghramian 2019; Chakravartty 2017). The relation of SP and ER has not yet been examined from the perspective of “stances.” Given the popularity of SP and card-carrying scientific pluralists’ occupation with demarcating themselves from ER, connecting this debate with the debate on “stances” will provide a novel contribution to this discussion. I will examine one pluralist and one relativist position as stances to provide a different perspective regarding their relation. I will show that differences in emphasis of VEPPs discern some relativist and pluralist positions. If particular emphases are sufficiently similar, it is possible to support ER and SP simultaneously. If VEPPs are too different, strong responses result. In a finalizing fifth step, I will connect the idea of “stances” with my discussion of RoJ and epistemic values. I will also provide some concluding remarks on how to make sense of this complicated relationship.

## Renouncing the renunciation of judgment

In this section, I will address one common worry of scientific pluralists with regards to ER: RoJ. Subsequently, I will argue that although probably worrisome, RoJ is not a component of ER. Several card-carrying scientific pluralists regard normativity as central to their claims. Science should be more pluralist; more approaches should be introduced (Chang 2012; Kellert et al. 2006). They fear they would have to give up on their normativity if they accepted ER because they fear that ER invites RoJ.

There is another problem regarding normativity and the loss thereof. Scientific pluralists are concerned that they would have to accept all theories as equal if they embraced ER. Defenders of SP often state that ER entails an “anything-goes” attitude regarding a plurality of theories. They are concerned that the epistemic relativist does not have the repertoires to judge between “good” and “bad” theories. Most scientific pluralists believe, however, that not all, but only some theories should be accepted. They are disinclined to regard all theories as equal. For example, several scientific pluralists believe that “creationism” should not get a seat on the table (Dupré 1993). Others aim to exclude research programs, which are excessively monist (Kellert, Longino, and Waters 2006).

Identifying ER with an “anything goes” attitude, that is, admitting all research programs to the table, rests on the notion of “equal validity.” “Equal validity” is one version of the “symmetry principle.” Card-carrying relativists define “symmetry” as a characteristic of their position (Bloor 1984; Ashton 2020). “Symmetry” is, first and foremost, the requirement to treat ES symmetrically. “Symmetry” can be construed in many different ways, and “equal validity” is only one of them (Kusch 2016; Ashton 2020). Critics of ER often argue that ER entails the belief that all possible epistemic systems (ES) are equally good. Because the epistemic relativist believes all systems are equally good, she does not judge between them.

In what follows, I will proceed in two steps. First, I will unpack the RoJ argument and show why the epistemic relativist does not have to “renounce judgment.” In a second step, I will discuss the “equal validity” principle in more detail and demonstrate why the relativist cannot embrace “equal validity.”

Critics of ER often assume that RoJ is part of a relativist position because card-carrying relativists maintain no “absolute” way of judging between different ES. Critics of ER thus fear that because the relativist believes that there is no “absolute” way of judging between ES, she has to suspend judgment altogether.

The relativist’s rejoinder to this argument is straight forward. There is no absolute way of judging between ES. Judgments are only possible from within a particular ES. There is, as a result, no way to provide a “gods-eye” perspective on ES, and therefore come to an “absolute” conclusion when assessing or ranking a plurality of ES. While the relativist stands by the rejection of “absolute judgment,” she nevertheless believes that (non-absolute) judgment is possible. The epistemic relativist believes that it is possible to pass judgment on one or several ES. She would, of course, declare this judgment as situated within the context of her ES. Nevertheless, the epistemic relativist can pass judgment and rank different ES by referring to her ES. To her, a statement does not lose its normative content once it is situated. Quite to the contrary, relativized assessments are all that can be. In conclusion, the relativist does not renounce judgment. She only renounces the possibility of absolute, neutral judgment (Feyerabend 1975/2010, p. 154; Barnes and Bloor 1982, p. 27; Code 1995, p. 203).

I will move forward and assess the pluralist’s second concern regarding ER: “equal-validity.” Symmetry is the guideline to treat ES symmetrically. There are different ways to motivate this guideline. One of them is “equal validity:” ES should be treated symmetrically because they all are equally valid. First of all, “equal validity” and RoJ are not compatible. If the relativist would renounce judgment, she could not proclaim “equal validity” of all ES. On the other hand, if the relativist would support “equal validity,” it would not be possible to support RoJ because “equal validity” is a judgment. In previous paragraphs, I have argued that the relativist does not support RoJ. In what follows, I will repeat the exercise regarding “equal validity.”

Many critics of ER believe that commitments such as plurality, dependence, and exclusiveness necessitate the equal validity clause. The symmetry clause fortifies this belief. Avoiding “equal validity” is, however, essential for many philosophers. For example, feminist philosophers of science reject relativism because they reject the “equal validity” clause. They argue that while they believe that knowledge is situated, not all perspectives (such as, e.g., the male chauvinist’s) are equally valid (Crasnow 2014, p. 154; Harding 1991, p. 139).

It is, however, essential to note that the relativist only discourages the ranking of systems when referring to absolute criteria (Barnes and Bloor 1982, p. 23). She acknowledges the exclusiveness of individual ES. Inhabitants of one ES might rank an ES that provides a conflicting (yes/no) answer to a particular question lower than their own ES. This is what the relativist expects. What she faults is ranking such systems in an absolutist sense. One of the most common ways to account for this notion is the “non-neutrality” clause: the belief that there is no neutral way of ranking ES.

While “non-neutrality” is the most common way to motivate symmetry, “equal validity” faces severe problems from a relativist point of view. Where would this perspective come from that allows for the judgment that all systems are equally valid? Acknowledging such a meta-perspective would point towards absolutism. But the relativist rejects absolutism, which is why a relativist cannot support “equal validity”.^6^

Let me illustrate my claim. A relativist might assess that a particular alternative ES is consistent and persuasive for actors within it. Yet she does not have to treat this other ES as equal to her own just because it is coherent or consistent (Kusch 2020, p. 143). To recognize the constitution or structure of an alternative ES as similar to one’s own does not provide reasons to convert to it (Rovane 2011, p. 46f). The relativist could still hold that her ES is better, with respect to her ES, because of the differences between these ES. Both systems could, for instance, rank specific values differently. While she believes her system to be better, with respect to her ES, the relativist would realize that she might not convince proponents of the alternative ES to convert to hers. This example describes the type of symmetry that is characteristic of ER. This type of symmetry does not entail equal validity and does also not entail RoJ.

## Epistemic values to the rescue?

In the previous section, I have sketched the scientific pluralists’ concern with ER. She fears that by adopting ER, she cannot judge between different theories. She believes that she might not be able to sort out the “foul fruits.” As I have shown, it is possible to reject this concern right away. Because the relativist does not and cannot support “equal validity,” one can refuse the pluralists’ arguments out of hand. Even though I have already undercut this concern, I will not presuppose my argument on equal validity and determine how the introduction of epistemic values^7^ fares in differentiating ER and SP.

How do pluralists preserve judgment? Most card-carrying scientific pluralists achieve this by introducing sets of values for executing theory choice. Only those theories that fulfill specific values can be kept in the pluralist framework. But, how are sets of values chosen? I will argue that introducing values does not set SP apart from ER. The introduction of values, instead, brings into view another level on which relativism applies. Relativism also applies to the level at which values are chosen.

Kuhn’s paper on theory choice prominently lists five values: “accuracy,” “consistency,” “scope,” “simplicity,” and “fruitfulness” (Kuhn 1977, p. 102f). Values, as proposed by Kuhn, are considered strictly epistemological or “cognitive” values. Other sets of values go beyond what is traditionally conceived as epistemological. Feminist values, for example, provide an epistemological perspective on how and what knowledge should be attained. Human beings have heterogeneous needs, and science, therefore, should address these needs. Such values, thus, have a political agenda. They are epistemological and “political.” These values determine how knowledge should be attained but also specify what kind of knowledge should be produced. “The feminist and traditional virtues are on a par, epistemologically. Both have heuristic but not probative power.” (Longino 2008, p. 74). For each context of inquiry one, or a selection of “traditional” and “political” values will be selected to guide investigations and yield the type of knowledge which is required in a particular context (Anderson 1995 and Longino 2008).^8^

Values proposed by feminist philosopher of science Longino are epistemological and political. In 2006, Longino co-authored, together with Kellert and Waters, the “Pluralist Stance” (PS). In their manifesto, they hold that pluralists, but not relativists, can pass negative judgment on “instances of bad research.” Kellert, Longino, and Waters do not spill much ink on which values they have in mind particularly. Thus, I will consider Longino’s discussion of values in her paper *Values, Heuristics, and the Politics of Knowledge* (2008).

Longino lists six feminist values that should guide theory choice. These values limit plurality by excluding “instances of bad research” (2006, p. xxvii). The first value is “empirical adequacy”: “agreement of the observational claims of a theory with data” (ibid.). Received and feminist sets of values share empirical adequacy. Longino emphasizes that empirical adequacy is not sufficient for theory choice. One of the classical problems regarding empirical adequacy is the underdetermination of theory by data. Underdetermination suggests plurality: more than one theory might be empirically adequate. The monist needs to resort to other values to limit the choice to one theory. The pluralist is content with a less restrictive choice, as she need not eliminate underdetermination but only keep out specific theories.

The additional values Longino proposes are novelty, ontological heterogeneity, complexity of interaction, pragmatic dimension of knowledge, and decentralization of power (2008, p. 70ff).^9^ Longino suggests her values as counterparts to conventional values. For example, novelty challenges conservativeness and consistency. Ontological heterogeneity challenges simplicity, parsimony, and unification.

Longino’s proposed values are a case in point for a set of values that might not be embraced by all philosophers of science or scientists. Some philosophers might insist on the superiority of the “traditional” values and prefer their realization over Longino’s values. For example, scientific pluralist John Dupré proposes epistemic values that resemble the traditional values closely.^10^ Longino’s pick of values is, of course, conditioned by a specific agenda: eradicating inequalities in science. There are, thus, principles that guide value choice. But who decides on these principles?

Most epistemic values are highly desirable. Yet one’s personal preferences and agendas define which values one gives priority. My review of two sets of values, the canonical and Longino’s feminist values, demonstrates the vast heterogeneity of values pluralists might put forward. If we look beyond the scientific pluralism literature and towards more “canonical” surveys of values, even more agendas are in view. A standard scientific realist account of values would, for example, prefer only those values that promote the truth of particular scientific theories (e.g., Haack 1996; David 2001).^11^ While a pluralist might regard truth conductivity as a beneficial outcome, she might prioritize other values and settle with “true enough” accounts (Elgin 2017; Potochnik 2017).

In some cases, these sets of values might be in tension; they might privilege different theories. Acknowledging this variety of values invites specific questions: Which values are to be adopted? How are values ranked? Does the choice of values happen randomly?

Of course not. Value choice does not happen randomly. There are other commitments or principles within a particular position that help rank particular values. If we characterize feminist epistemology as a rebellion against sexist theories, then the choice of values is part of the policy to change or exclude androcentric theories. If pluralism is a rebellion against monism, then the choice of values is part of the policy to challenge monity and monism in science. If the scientific realist aims for the truth of a theory, that theoretical statements about the world have face value, then her choice will fall on values that are truth-conductive. Also, for the standard definition of scientific realism [as opposed to, e.g., Hasok Chang’s “active realism” (2012)], she will choose values that help her choose one theory and not several.

Value choice does not happen randomly. But there is no non-circular way to motivate value choice. A broad and overarching set of commitments of what is believed to be “good,” “right,” “just,” or “fruitful” motivates value choice. If we value pluralism, we will select values that promote plurality. In other words, value-choice happens based on what we value in general, how we imagine science at its best, how we believe the scientific community should strive. These commitments justify our value choice, and our value choice feeds back on our commitments. Values are not chosen randomly, but there is no non-circular way to motivate one’s choice of values.

In conclusion, values are a crucial part of theory choice, and in combination with other commitments, choice is undoubtedly possible. Referring to “other commitments” brings me back to a more general point regarding the relativity of values and my argument regarding non-neutral judgment in the previous section. Assume an advocate of a particular position commits to specific values, yet a proponent of another position commits to others. Even though the former advocate might be able to reconstruct the choices of the latter, this does not provide grounds for adopting the second advocate’s values. The pluralist does not have to suspend judgment because she realizes that she might not convince a particular person regarding her preferred choice of values, and as a result, her choice of theories. But this does not make values any less worthwhile. Values are a crucial tool for theory choice. As there is an intrinsic relativity to values, they do not exorcise ER.

## Entailments, entailments… scientific pluralism and epistemic relativism

In the previous sections, I tried to demonstrate that the most common concerns scientific pluralists raise against ER can be smoothed and that the most common strategy of setting apart SP and ER fails. It is thus necessary to restate the leading question of this article: What differentiates SP and ER? And why are scientific pluralists’ responses against ER that forceful?

In this section, I will argue that there is no one-dimensional solution to this problem. I will show that there are some versions of ER a scientific pluralist would have to reject. But there are also some versions of SP, an epistemic relativist would have to reject. I will demonstrate that any general statement of the kind “X entails Y” is not applicable when talking about the relation of SP and ER.

Let me start with instances of pluralism a relativist would have to reject. Take the symmetry principle, for example. The pluralist could violate any form of symmetry (5a–5e). Let us consider, for instance, a normative pluralist position. Symmetry might be negligible to the normative pluralist. She could maintain that there is one, single, and absolutely best ES/approach. But she could still cherish the benefits of having more than this one. For instance, she could point to certain benefits of keeping more than one system or approach around. Chang’s benefits of tolerance and interaction would be famous examples of such benefits (Chang 2012, p. 279f). The relativist could also argue that although some other ES/theories are worse than others (in an absolute sense, even), they still capture exciting aspects of the world.

Another example is “exclusiveness.” Some versions of SP do not consider the presence of several ES. Such versions of SP describe theories or explanations within a framework considered one ES (take, Mitchell 2003, or Potochnik 2017 as examples). But even if a pluralist considers the existence of more than one ES, exclusiveness might not play a role in her position. The pluralist might argue for integration across ES. For example, Chang emphasizes ad hoc integration across “systems of practice” (Chang 2012).

Let me proceed and examine examples of ER a pluralist would have to reject. Although the pluralist rejects monism, she does not have to reject absolutism. For instance, she could utter the claim that it is absolutely true that SP is the superior way of gaining knowledge. It is possible, thus, to be an absolutist pluralist. A pluralist might reject versions of relativism that argue that ES should be treated symmetrically. This might obstruct the pluralist’s agenda. SP usually accounts for many different approaches, and ranking might be crucial.

Besides, the relativist accepts “plurality” but not necessarily “pluralism.” It is possible to be a relativist monist. The pluralist would have to reject versions of relativism that are monist. For instance, the epistemic relativist could have a negative attitude about a plurality of different ES.^12^ The relativist can, of course, pass negative judgment about a situation of plurality relative to her ES.^13^ She could also argue that within her ES, only one theoretical account should be maintained. A card-carrying scientific pluralist would need to reject such versions of ER. Lastly, the pluralist would also need to reject forms of relativism that violate “acceptance.” For the relativist, the statement “plurality is highly desirable” has different truth values in different ES. For the pluralist, the benefits of pluralism are absolute.

In conclusion, the pluralist needs to dismiss those relativist positions that interfere with certain aspects of the pluralist’s agenda and vice versa. Differentiating ER and SP by referring to necessary and sufficient criteria is thus not entirely successful. This section, however, revealed that it seems more successful to look at particular agendas of ER and SP, respectively, and thereby examine possible differences and points of friction. In the next section, I will examine which VEPPs are shared by both positions and which are not. I will assess whether focusing on emphases regarding VEPPs, rather than on necessary and sufficient criteria, will better elucidate the relation of SP and ER.

## Stances: relativism and pluralism reconsidered

In his 2002 book “The Empirical Stance,” Bas van Fraassen proposes a new way of understanding philosophical positions. He criticizes “principle zero,” that is capturing positions by a list of dogmata they embrace:

Principle zero: iff someone takes position X, she must believe or decide to believe a statement X + , which exists for every position X.

Rather than “principle zero,” embracing a particular position means to commit to bundles of values, emotions, policies, and preferences (VEPPs). Van Fraassen claims that one should understand philosophical positions as stances. A set of VEPPs characterizes particular stances.^14^ In the previous section, I have claimed partial success for comparing necessary and sufficient criteria of ER and SP to differentiate both positions. I concluded that points of friction crystallized by comparing these criteria hint to differences regarding agendas and policies.

This section aims to investigate whether framing ER and SP as stances provides better clues regarding these positions’ differences and similarities. In so doing, I will survey their respective VEPPs. First, I will assess the possible benefits and drawbacks of this approach. If van Fraassen is right, conceptualizing positions as stances helps to understand the core of these positions, distill differences, and crystallize disputes between stances. As the focus lies on commitments such as values, policies, and emotions, the approach might reveal disagreements that do not play out fully when analytically comparing two positions but cause friction in practical settings. It is, however, not clear why one should focus on VEPPs and not on the very credo or dogma of a position (Kusch 2020).^15^ Whether a particular position is better captured by understanding it as a stance or as a position is, therefore, a matter of empirical assessment.

I will select one version of ER and one version of SP to capture them as a stance: I chose David Bloor’s Sociology of Scientific Knowledge (SSK) as a paradigm approach for ER (summarized in Table 1). Other versions of ER, for example, Lorraine Code’s feminist relativism (1995) or Paul Feyerabend’s epistemological anarchism (1975/2010), have recently also been characterized as stances (Baghramian and Coliva 2019). Proceeding with SSK is, however, the most convenient choice because Kusch has recently analyzed SSK for its VEPPs (2020). I will compare SSK to the “pluralist stance” (PS), a position defended by Longino, Kellert, and Waters. I take these two positions as good exemplar cases for understanding points of friction between ER and SP. PS explicitly rejects ER and refers to Bloor. I thus perform my analysis on a couple of positions with a report of friction in the literature. The goal is to understand the roots of the disagreement^16^ and tentatively project the results to a more general level.

**Table 1:** Assessing SSK (Bloor) and the Pluralist Stance (Longino) as stances to map commonalities and differences

	SSK (Bloor)	Pluralist stance (Longino)
Admires	Science (social science), plurality	Science (natural and social science), plurality
Policy	Four tenets of the strong program	Empirical commitment, feminist values
Faults	Epistemology and philosophy of science for lacking controlled data input	Androcentrism, “equal validity,” monism without empirical testing, demolishing opponents
Motivation	Results of empirical case studies	Results of empirical case studies
Encourages	Epistemic humility, tolerance, equality	Tamed realism, openness to plurality, equality
Rejects	Absolutism (metaphysics, epistemology, or ethics that posit absolutes), “weak program”	Fundamentalism and monism (metaphysical prejudice), “modest” or “radical” pluralism
Emphasizes	Group processes (“normative rationality”)	Group processes (and the diversity of them)

Kusch argues that SSK is an excellent candidate to be analyzed as a stance because SSK emphasizes VEPPs rather than a specific doctrine. Bloor’s central doctrine, “there are no context-free or super-cultural norms of rationality,” should be understood as a rebellion and foregrounds the policies SSK emphasizes. SSK highlights four policies to guide research: SSK emphasizes how social and other causes bring about belief. SSK urges for impartiality concerning rationality and irrationality, success and failure, truth and falsity. SSK scholars take the same interest in both sides of these dichotomies. SSK prescribes symmetry: the same types of causes explain both failure and success, truth and falsity, as well as rationality and irrationality. Finally, SSK requires reflexivity: the program applies to itself (Bloor 1984). SSK rejects the “weak program,” which maintains the dichotomies SSK rejects. Approaches considered part of the “weak program” explain failure but not success by referring to sociological reasons (Kusch 2020).

Bloor argues that the policies SSK emphasizes are resonant with how the empirical sciences operate. SSK thus admires the sciences, especially the social sciences. SSK takes an interest in the plurality of different theories, paradigms, or epistemic systems. This plurality informs case studies on disputes, episodes of plurality, within science. The results of these case studies motivate SSK scholars. Conversely, SSK faults epistemology and philosophy of science for lacking a similar type of controlled data input (2019).

SSK policies resonate with the value of epistemic humility: we should not lay universal claim to rationality or truth. Epistemic humility can be understood as a rebellion against any form of absolutism in metaphysics, epistemology, or ethics. Besides epistemic humility, SSK encourages tolerance and equality. SSK emphasizes group processes as explanatory for the phenomena described: there are different codifications of our reasoning properties, defined by interests and negotiations of different groups. SSK scholars summarize this doctrine as “normative rationality.” (summarized in Table 1).

In what follows, I will discuss PS for its eligibility as a stance. PS seems quite eligible because the authors restrain from providing particular necessary and sufficient criteria for the pluralism they advocate. Besides, Kellert, Longino, and Waters call their position a stance.

Proposing a particular policy is one of the most significant trademarks of a stance. In the case of PS, the authors propose two types of policies. On the one hand, there is the doctrine of “empirical commitment.” Kellert, Longino, and Waters argue that it has to be empirically assessed whether pluralism is the right interpretation of a particular situation (2006). Thus, they reject any metaphysical predispositions regarding pluralism but opine that adopting pluralism is a case-by-case matter. On the other hand, the feminist values Longino proposes play an essential role as a policy. These values help guide which theory should, in principle, get a seat on the table if SP is an adequate interpretation of a given situation.

The authors of PS have a positive attitude regarding plurality and admire cases in which it is impossible to settle on one best theory. They are both intrigued by instances of plurality in the natural, as well as in the social sciences. What motivates their program and its empirical commitment are the results of the empirical case studies they discuss (2006). Also, they focus on scientific endeavors as group processes and do not highlight the work of individuals. Besides, they emphasize the vast diversity of group processes within science.

As pointed out above, PS suggests the policy of empirical testing. As a result, the authors of PS reject particular philosophical positions, especially fundamentalism and monism, if these are founded on metaphysical prejudice. They also reject specific versions of SP. On the one hand, they reject “modest,” SP. “Modest pluralism” holds that it is possible to reconcile various approaches in one way or another. On the other hand, they reject “radical” pluralism, that is, pluralism that is anti-realist, or pluralism that supports an “anything goes” vibe. The authors of PS fault the “equal validity” thesis, and they fault demolishing opponents based on metaphysical prejudice (monism) (2006). Longino, in addition, faults androcentrism and other behaviors that promote inequality.

PS favors a “tamed realism.” Kellert, Longino, and Waters discourage strong metaphysical presuppositions regarding what the nature of the world is like and whether any of our positions, be it monism or pluralism, can answer this question (2006). They encourage an in principle openness towards plurality and openness to empirically assess whether SP is an adequate interpretation. Besides, they encourage equality, as realized by the adoption of Longino’s proposed values (summarized in Table 1).

Analyzing both SSK and PS as stances (see Table 1) highlights their similarities and differences. Given that the authors of PS, and Longino in particular (2002), reject any form of relativism, it seems surprising that both stances share quite some similarities. I will first analyze what both stances have in common and then focus on their dissimilarities. One particular commitment that spans across their VEPPs is both stances’ emphasis on empirical data. SSK and PS are motivated by empirical case studies. SSK faults the philosophy of science for lacking controlled data input. PS faults particular positions within the philosophy of science that are grounded on metaphysical presupposition and do not consider empirical evidence. Also, both stances share an admiration for plurality and emphasize group processes over individualism.

Both stances promote partly similar, partly different values. Both champion the establishment of equality. While SSK lays a particular focus on epistemic humility and tolerance, the pluralist stance emphasizes (feminist) empiricism. Both stances engage in boundary work to demarcate their position from related positions. SSK strongly emphasizes its rejection of the “weak program,” whereas PS rejects “modest” as well as “radical” pluralism. While SSK rejects absolutism, PS rejects fundamentalism, monism, and relativism (explicitly referring to SSK). To be precise, PS rejects the “equal validity” thesis of relativism.

One key difference is the proposed policies and the level on which policy applies. SSK’s policy determines how to do social science. PS’ policy operates on the meta-level (how should philosophers think about science) and on the level of the empirical sciences (which approaches should be pursued by researchers). In this sense, it is possible to discern a difference in the levels of analysis. SSK is a doctrine on how to collect and analyze data regarding particular scientific episodes and which types of explanatory strategies should be employed. PS demands the philosopher to have a particular outlook on the empirical sciences and also requires a similar commitment of scientists: PS demands the introduction of more approaches and empirical assessment of these approaches. This task will not lie on the shoulders of philosophers alone. From the perspective of the SSK-er, the absolutist claim that scientists and philosophers should adopt a particular epistemological position is a no-go. For the defender of PS, discouraging the PS’s normative approach towards changing scientific inquiry means to negate the policy that is dearest to proponents of PS.

In conclusion, portraying SSK and PS as stances highlights similarities and differences. SSK and PS share a commitment to empirical input, particular virtues, an admiration for science and plurality displayed in science. PS’ rejection of “anything goes” needs to be mentioned as a difference. Having shown, however, that the “anything goes” argument is a strawman, I do not believe that this rejection should receive center stage regarding the aspects that differentiate both positions. It thus does not come down to principled rejections of specific necessary and sufficient criteria. The most noticeable difference is the particular policies SSK and PS champion. These different policies make visible the different emphases of the respective stances and what, exactly, each stance’s proponents would like to see changed about philosophy, sociology, and science. It is this disagreement about policy and the level on which policy should apply that causes real-life friction and the difficulties individual proponents of PS and SSK might have with each other.

Analyzing SP and ER as stances or analyzing them as done in the last section leads to partly similar, partly different results. There is no reason why SP and ER would not go together in principle. To combine both ER and SP in one’s position, certain VEPPs need to be sufficiently similar. Paul Feyerabend’s early and late epistemological anarchism is an example of a position that admits both SP and ER (Kusch 2016). There are also more recent examples. In *Disorder of Things,* John Dupré proposes a version of pluralism that places purpose-sensitivity (promiscuous realism) as its central motivation (1993). As a result, his SP also admits ER because Dupré’s ranking of VEPPs is sufficiently similar to ER’s ranking of VEPPs. It is thus possible to commit to both ER and SP. This depends on particular emphases and ranking of VEPPs.

What becomes clear through understanding SP and ER as stances is that SP and ER have a different agenda most of the time. ER and SP commit to partly similar and partly different VEPPs. For SSK and PS, what differentiates both stances most, is a difference in policy. Deep disagreements about a particular policy cause strong responses of scientific pluralists against ER in the literature. These policy differences are, however, seldomly addressed, and arguments for repudiating ER such as RoJ or “equal validity” are invoked instead.

Whether SP and ER “go together” in one philosopher’s position is thus a matter of emphasis and ranking of particular VEPPs. Take philosopher and historian of science Hasok Chang as an example. In earlier works, Chang emphasized his rejection of relativism (2012, 2015). If one looks at more recent contributions, Chang has shifted his argument to the idea that relativism is “passive,” and pluralism is “active”^17^ (2020). That is, Chang disagrees with ER only in so far as he deems the policy of ER to be unfit for his purposes. Thus, Chang’s discontent with ER comes down to a different ranking of particular VEPPs.

This brings back a more general notion about the difference between SP and ER: the level of analysis. SP usually characterizes positions that operate on the level of the empirical sciences [e.g., Chang’s appeal for a re-appreciation of phlogiston theory (2015)], while epistemic relativists often intervene on the meta-level of philosophical analysis [e.g., Kusch’s critique of how Chang reached his conclusions about the chemical revolution (2015)]. This differentiation was also apparent when comparing SSK and PS. While I disagree with the idea that ER has to be “passive,” it seems safe to say that particular preferences that are more likely to be found within ER stances make ER often appear more “passive” or operate on the meta-level exclusively.^18^

## Conclusion

Introducing the idea of the “stance” and “VEPPs” also weighs in on the relativist’s rejection of RoJ. Different ES might share certain VEPPs but might not share others. Proponents of different ES will rank some of the shared VEPPs differently in importance. In a situation when the epistemic relativist compares her ES with another ES, she will discover that certain VEPPs her ES ranks high might not be that important in the other ES. On the other hand, she will find out that the other ES ranks particular VEPPs high, which she considers less important.

If she values a particular policy but sees this policy not valued in a different ES, why would she say “whatever” (Chang 2012)? The fact that VEPPs are ranked differently will give her reasons to prefer her ES over the other, with respect to the VEPPs of her ES. But because she realizes how both ES are constructed and that an intricate connection of different VEPPs accounts for the differences, she will also realize that she might not convince proponents of the other ES to convert to her ES.

The choice of possible VEPPs is vast. I argued that some of these might contradict each other or cannot be embraced simultaneously. Thus, there needs to be a choice—choice of a particular set of VEPPs over another. I pointed out that this choice is informed by other commitments, for example, in the case of feminist philosophy, the policy to rid science of androcentric theories (Harding 1992). In the case of PS, proponents commit to avoiding monism and introducing a plurality of theories. To reformulate what was argued in a previous section, now taking the idea of VEPPs into account: value choice is governed within a broader context of VEPPs. Not only that, values, emotions, policies, and preferences inform each other.

Viewing one’s choice of epistemic values as part of one’s stance also brings home the idea of these values’ intrinsic relativity. The defender of one particular assortment of VEPPs might realize that the assortment of, say, another scientific pluralist does not resonate with hers. She would, of course, not believe that she cannot pass judgment or believe that both sets of VEPPs are “equally valid.” Take the feminist philosopher, for example. Why on earth would she believe that a particular system that does not rank specific values highly, that she believes are crucial for criticizing androcentric theories, is as good as hers, with respect to her system? She would, of course, also believe that it would be better if that other system ranked values differently. But she will also realize that she might not be able to convince a proponent holding on to a different ranking of VEPPs.^19^ Epistemic values are relative, but this does not mean that negative judgment cannot be passed regarding particular ES. Judgment can always be passed, but from within one’s ES. The relativist does not support RoJ, but she renounces neutral judgment. To the relativist, judgments dependent on a particular ES are all there is, given the “non-neutrality” clause.

In this article, I aimed to explore the relation between SP and ER and understand points of friction. I believe that I have shown several things. First, RoJ is neither part of ER nor of SP. Second, the introduction of epistemic values guides theory choice but cannot set apart SP from ER because it introduces relativism on a different level. Third, SP does not necessarily entail ER and vice versa. Fourth, applying the perspective of the stance, similarities and differences of both positions become discernable. If emphases are sufficiently similar, it is possible to support ER and SP. It is possible to be a relativist and a pluralist at the same time. Being a relativist pluralist is not epistemologically pernicious. It determines a particular stance, with particular choices of VEPPs. Disagreements about emphasis and policy set some epistemic relativist positions apart from some scientific pluralist positions. Disagreement about such policies brings about strong responses against ER. While there are no principled reasons why SP and ER cannot go together, policy differences often prevent accepting both positions in practice.

## Footnotes

1 Perspectivism is also a position which, as does SP, enjoys great popularity amongst philosophers of science. The examination of the relation of ER and perspectivism (Giere 2006; Massimi 2017) requires a separate discussion. At this point, it is however worth mentioning that questions about the relation of SP and ER and the relation of ER and perspectivism might be quite similar (this issue is primed, for example, in Chang 2020).

2 Apart from the discussion regaring scientific pluralism and epistemic relativism, there is also a lively debate regaring the relation of epistemic pluralism and epistemic relativism (e.g. Coliva and Pederson 2017). Epistemic pluralism addresses the debate on what is justified true belief. There are several conditions proposed as necessary for justification, amongst them truth-conductivity, cognitive accessibility, coherence and the like. William Alston is a prominent defender of epistemic pluralism. Rather than searching for the overall best condition, Alston proposes that all these conditions are epistemically highly desirable (Alston 1993, p. 532). Thus, these conditions are to be seen as “epistemic desiderata”. The choice of the respective desideratum (or desiderata) might be different for each context (Alston 1993, p. 534). Similarities and differences of ER and EP have been addressed in Kusch (2017a, b) and Carter (2017).

3 In certain versions of this definition, “plurality” is exchanged for “pluralism” (Kusch 2017a, b). In these cases, the qualification: “there are, were or might be several of such ES or bundles,” is maintained to describe “pluralism.” I suggest that such definitions of pluralism (also to be found in Boghossian 2007), should be read as denoting plurality, not pluralism, as no positive attitude with regards to plurality is indicated.

4 Symmetry is where critics of ER locate RoJ. In sections to follow, I will argue that “equal validity,” as expressed in 5c, is a commitment that no card-carrying relativist supports. Symmetry is usually realized by committing to either 5a, 5b, 5d, or 5e.

5 I added 5e to Kusch‘s 5a–5d, to account for the symmetry principle in the social sciences. Supporting 5e does not require to support any of 5a–5d.

6 The rejoinder of the “equal validity” charge provided here turns the “classical” arguments against relativism on their head (Siegel 2011). While critics of ER argue that ER refutes itself, because claims such as “everything is relativist” or “equal validity” are absolutist theses, I argue that the epistemic relativist has to reject “equal validity,” exactly because it requires an absolutist perspective.

7 In this section, and the remainder of the paper, I will explicitly refer to values (as in characteristics a theory realizes), and not virtues (as in particular charcter traits of a researcher/philosopher/individual). Several scientific pluralists, however, go into length in proposing virtues of scientists, or philosophers of science. By realizing these virtues (e.g. humility, prudence, curiosity…) these researchers foster plurality as a result. Such elaborations can be found in Chang (2012), Feyerabend (1975/2010), also discussed in Kidd (2016). It is also important to note, that also defenders of relativism believe that ER cultivates particular virtues (Baghramian 2019).

8 It is important to note that also “traditional” values, have political valence if put into a particular context. Take values such as “simplicity” and “uniformity.” Until the early 1990s, effectiveness and appropriate dosages of medications were tested only in white, male cohorts. Feminist values, such as “ontological heterogeneity” require testing that is sensitive to the heterogenous set of individuals, such as women, non-white men, and non-cis men and women. But it is not the case that only the “challenging” value, “ontological heterogeneity” is “political.” Insisting on the values of “uniformity,” or “simplicity,” to guide a particular research trial, also is Longino (2008).

9 “Novelty” allows breaking with established theoretical frameworks. Novelty is particularly important when it helps to choose theories that break with traditional sexist theories. “Ontological heterogeneity” specifies the content of a theory. In order to satisfy ontological heterogeneity, a theory needs to propose different kinds of entities and causal factors. If theories are ontologically homogenous, they might miss significant differences between causal agents. Feminists mainly criticize that males are often treated as “the only causally effective agents in a population” (2008). The “complexity of interaction” defines how interactions between entities should be defined. Interactions should be represented as complex, mutual, and reciprocal (ibid.). Theories that do not reduce complex processes to simple causal structures are preferred. The “Pragmadtic dimension of knowledge” defines that knowledge should be for people. For example, research that alleviates the needs of women should be preferred over research for military purposes or research for knowledge’s sake only. “Decentralization of power” determines that: “Forms of knowledge and its application in technologies that empower beneficiaries are preferred to those that produce or reproduce dependence relations” (ibid.).

10 These values include “sensitivity to empirical fact, plausible background assumptions, coherence with other things we know, exposure to criticism from the widest variety of sources” (1993).

11 This is not to say that scientific pluralists do not support realism. Chang (2012, 2018) for example, proposes a particular form of realism, that resonates with his version of pluralism. It is nevertheless important to say that pluralists might value truth conductivity, this value has not a sina qua non position within the pluralist framework.

12 As discussed in footnote 3, definitions of relativism (Kusch 2017a, b; Boghossian 2007) that list “pluralism” as a characteristiv of ER provide a definition of “pluralism,” that denotes “plurality,” but not “pluralism.”.

13 There are examples, where passing negative judgment also extends to other ES. In his critique of Chang’s scientific pluralism regarding the chemical revolution, Kusch seems to pass negative judgment regarding plurality. Pointing to the experimenters’ regress, he argues that actors were justified in abandoning phlogiston theory (2015).

14 Van Fraassen exemplifies his idea on stances by taking “empiricism” as an example: “Being an empiricist cannot consist in believing some statement about what the world is like.” (Van Fraassen 2002, p. 47). One should understand empiricism as a rebellion against metaphysics and capture this position by accounting for the value judgments empiricists embrace. Van Fraassen lists the following VEPPs for empiricism (E): E rejects the demand for explanations by postulate, E recalls to experience, E rebels against theory, E idealizes epistemic rationality, E admires science… Van Fraassen concludes: “A philosophical position can consist in a stance (attitude, commitment, approach, a cluster of such—possibly including some propositional attitudes such as beliefs as well)” (2002, pp. 47–48).

15 There is a circular relation between VEPPs and dogmata. Kusch maintains that specific VEPPs inform dogmata and these dogmata necessitate specific VEPPs. It is to be assessed empirically, whether advocates of a specific position foreground a doctrine or whether they emphasize VEPPs.

16 While I regard SSK and PS as exemplary cases that help understand frictions between ER and SP, I do of course admit for cases that do not show the same frictions. Below, I will hint at particular pluralist positions, such as Feyerabend’s or Dupré’s which admit for ER and SP.

17 At this point I would like to express my opinion that I do not believe that relativism is “passive,” as opposed to SP, which is active. How a relativist could be active, and also uncover new systems of practice, to keep with Chang’s terminology, needs to be discussed in a different paper.

18 It is nevertheless possible for the epistemic relativist’s investigations (e.g. of an SSK or STS type) to address the empirical sciences themselves (Jasanoff 1996).

19 Ashton (2019) makes a similar point about the relation of Standpoint Theory and Epistemic Relativism.
